# A Microvascular System Self-Healing Approach on Polymeric Composite Materials

**DOI:** 10.3390/polym14142798

**Published:** 2022-07-08

**Authors:** Ionut Sebastian Vintila, Jana Ghitman, Horia Iovu, Alexandru Paraschiv, Andreia Cucuruz, Dragos Mihai, Ionut Florian Popa

**Affiliations:** 1 National Research and Development Institute for Gas Turbines COMOTI, 220D Iuliu Maniu Avenue, 061126 Bucharest, Romania; alexandru.paraschiv@comoti.ro (A.P.); dragos.mihai@comoti.ro (D.M.); ionut.popa@comoti.ro (I.F.P.); 2 Advanced Polymer Materials Group, University Politehnica of Bucharest, 1-7 Gh. Polizu Street, 011061 Bucharest, Romania; jana.ghitman@upb.ro; 3 Department of Bioresources and Polymer Science, Faculty of Chemical Engineering and Biotechnologies, University Politehnica of Bucharest, 1-7 Gh. Polizu Street, 011061 Bucharest, Romania; horia.iovu@upb.ro; 4 Department of Science and Engineering of Oxide Materials and Nanomaterials, Faculty of Applied Chemistry and Materials Science, University Politehnica of Bucharest, 1-7 Gh. Polizu Street, 011061 Bucharest, Romania; andreia.cucuruz@upb.ro

**Keywords:** composite, self-healing, microvascular, nanofibre

## Abstract

The paper addresses the synthesis of a nano-fibre network by coaxial electrospinning, embedding the healing agent dicyclopentadiene (DCPD) in polyacrylonitrile (PAN) fibres. Compared to other encapsulation methods, the use of nano-fibres filled with healing agent have no effect on the mechanical properties of the matrix and can address a larger healing area. Additionally, carbon nanotubes were added as nanofillers to enhance the reactivity between DCPD and the epoxydic matrix. The self-healing capability of the nano-fibre network was carried out by flexural tests, at epoxy resin level and composite level. Results obtained from Fourier transform infrared (FTIR) spectrometry, thermogravimetric analysis (TGA) and scanning electron microscopy (SEM) confirmed the successful encapsulation of DCPD healing agent in PAN fibres. Flexural tests indicate that after 48 h, the epoxy resin has recovered 84% of its flexural strength while the composite material recovered 93%.

## 1. Introduction

The polymeric composite materials (PCM) took a lot of attention from important industries (space, aerospace, automotive, military) as a great alternative for existing structural metallic materials, mainly by using thermoset epoxydic matrix reinforced with carbon fibres. The obtained carbon fibre reinforced polymers (CFRP) have a lightweight, high strength structure, considering its physical and mechanical properties (high strength-to-weight ratio, high modulus, design adaptability, flexibility to various geometries, great fatigue and corrosion resistance, good thermal expansion properties). As the CFRP provides many advantages their matrix has a brittle nature, and when different mechanical loads are applied (vibrations, stress, tension, etc.), it promotes cracks and delaminations that lead to losing their structural integrity.

Generally, the failure modes occur at the nanoscale level and propagates to micro and macro levels, and this affects more or less in time the rate at which the material yields to the loads to which it is subjected to [1]. To this, different methods were used to aid the healing of the material, starting from the nano and micro level, thus stalling or stopping the crack from propagating further and affecting the integrity of the structure. Considering that the budget for repairing damaged composites is growing year by year [2], repair processes are time- and cost consuming. Having this, the addition of self-healing methods is appraised by many industrial companies and scientific community. Basically, by using their self-healing ability, polymeric materials can transform the physical energy (from the damage) into chemical energy required to heal the affected area [3,4,5]. In this regard, different healing agents and encapsulation methods have been studied and reviewed [6,7,8,9].

As the microcapsules are the most used and versatile self-healing method, they have an extremely tedious preparation process and also limit the potential healing applications. In comparison to microcapsule healing process, the use of microvascular self-healing systems permits multiple healing processes, allowing the healing agents to be spread and reach distant points, covering a larger area [10].

Also, as seen in the previous work performed by the authors [11,12], even though the microencapsulation of healing agents leads to an 80–85% recovery of mechanical properties, the integration of microcapsules within the matrix and the composite material acts as an induced damage and thus reduces the mechanical properties of the specimen, as compared to the reference ones. This is mainly due to the microcapsule dimensions and volume used, and the fact that after breakage, the microcapsule shells are left within the material, further acting as a stress concentrator for other mechanical loads to which the structure can be subjected to. To reduce these shortcomings, the authors have proposed the use of nano-fibres fabricated by coaxial electrospinning process, that act as a microvascular network within the matrix and the composite material, respectively.

Initially demonstrated in 2010 by Braun et al. [13], the production and integration of nano-fibres self-healing materials within the composite material have shown that it has little to none influence on the mechanical properties of the matrix or the composite material. Having a tangled aspect, the nano-fibre mats can deliver the encapsulated agent into the affected area in a more rapid way. Depending on the fibres used as shell materials, these nano-fibres have the possibility to increase the mechanical properties of the matrix or the composite material itself [14].

Comparing the three known encapsulation methods, the development of nano-fibres through the electrospinning process is shown to be more cost-efficient solution to cover a larger, multiple healing area due to its random entanglement of nanofibers. Another major advantage of the electrospinning process relies in the controllability of the process to obtain a higher surface-area-to-volume ratio and the formation of a homogenous structure with no defects [15].

Different core-shell nano-fibres have been conducted to extend the lifetime and mechanical performance of different composite laminates, by incorporating healing agents into the core, inducing the healing ability to the composite structure. In 2012, Sinha-Ray et al. [16] have encapsulated dicyclopentadiene (DCPD) as a healing material in polyacrylonitrile (PAN) shell material using emulsion electrospinning. The authors evaluated the self-healing ability by introducing the core-shell nano-fibres within the composite layers and subjected the specimens to interlaminar fracture tests, demonstrating the release of the healing agent and facilitating its solidification within the crack. Wu et al. [17] have encapsulated DCPD in PAN nano-fibres by means of coaxial electrospinning, incorporating them together with Grubbs catalyst within CFRP layers. To assess the self-healing ability of the core-shell nano-fibres, the authors performed three-point bending tests and the results showed flexural stiffness values after healing of about 89% in average.

In the work carried out by Neisiany et al. [18], the authors have studied the self-healing ability of a CFRP composite by incorporating an epoxydic resin in Styrene acrylonitrile (SAN) nanofibers structure by means of coaxial electrospinning method, and placing the core-shell nanofibers between CFRP layers. Having a high encapsulation yield (>90%), mechanical tests showed no reduction of mechanical properties for the composite embedding the core-shell nanofibers, and allowed the composite to have up to three repair cycles (97%, 94%, and 89% recovery), until exhaustion of healing agent. Other studies have been performed by Neisiany et al. [19,20,21] and successfully shown the releasing and solidifying of the healing agents using PAN and poly(methyl methacrylate (PMMA) as shell material.

Mohammadi et al. [22] studied the self-healing capability of a glass fibre reinforced polymer composite (GFRP) having an epoxydic resin as healing agent within the microvascular channels and embedded them in the composite. Mechanical tests and microstructural analyses have shown that the healing ability was confirmed at different periods of time, namely after 4, 7, and 11 days, receiving a healing efficiency of maximum 69%, compared to the reference specimen.

Polymer nanofibres have a great potential in many applications, as filtration material, biomedical, material reinforcement and electronics. To ease the manufacturing process of nanofibres, electrospinning processing technique has been widely used. Depending on the application, different nanofibres surface properties are desired, including wettability, adsorption and adhesion. One of the methods to improve the surface properties of the electrospun nanofibres is the cold gas plasma treatment, that modifies the surface of polymer nanofibres without affecting their bulk properties [23,24]. In the work carried out by Wei et al. [23], cold gas plasma treatment was used to analyse its effect on surface properties of polyamide 6 (PA6) nanofibers. It was observed that by changing the PA6 surface properties it has enhanced the surface roughness of the nanofibers and also, the contact angle between water and nanofibers has been reduced significantly. The authors concluded that the nanofibres surface modification has a great potential in biomaterials, sensors and medical devices applications. Another method of improving the surface properties of the electrospun nanofibres is the air plasma functionalization, which improves the hydrophilicity of the nanofibers. Considering bio-medical applications, Mozaffari et al. [25,26] have studied the effect of atmospheric air plasma method and Argon-Oxygen plasma surface modificaiton on improving the bio-functionality of the electrospun naofibres. As also reported by [23], the surface roughness has been significantly increased due to ionization and chemical degradation process. Also, the hydrophilicity, biocompatibility and bioactivity were improved.

Although the electrospinning process of developing nano-fibres are mostly used in medical and biomedical applications, the nano-fibres developed through electrospinning process has taken large interest for other applications, as in the self-healing polymers. This is due to the large variety of materials that can be used to develop nano-fibre, as thermoplastic and thermosetting polymers, conjugated polymers, natural polymers, metals, metal oxides, nitrides and carbides, etc. There are many types of electrospinning processes depending on the final application, single fluid process, multiaxial, coaxial, triaxial, multi-needle, etc. [2,27,28]. A single-fluid blending electrospinning process was used together with casting film method to develop nanofibres for drug-controlled release application [29]. This combination between electrospinning and traditional casting method for producing functional hybrid films has been demonstrated successfully, providing a dual-phase drug-controlled release. This technique can be applied also in the self-healing application for polymer composites, where a controlled release of healing agent can benefit the healing efficiency. A side-by-side electrospinning technique has been used by [30] to develop a nanofiber wound dressing to promote the wound healing process, comprising three layers. The technique can be used in self-healing polymers applications, where both the healing agent and the catalyst can be embedded in the same nanofibre, separated by a middle layer. Same as side-by-side process, a tri-fluid electrospinning techniques can also be applied to deliver the healing agent into the polymer crack, to prevent propagation. Such technique was performed to develop tri-section nanofibres, to deliver a poorly water-soluble medicine [31]. By introducing nanofiller reinforcements such as carbon nanotubes (CNTs) in the polymeric matrix, not only it enhances the mechanical properties of the material, but it also offers structural, electronic and thermal properties which, in the end, may be beneficial for the healing ability of the self-healing system. More precisely, depending on the industry and application, the electronic advantage given by the CNTs may activate the healing agents when trapped within the crack, generated by small electric impulses. As aircrafts have many electronic controls that act on different composite components, the use of these impulses together with the CNT nanofillers may increase the healing ability of the corresponding components. Different self-healing polymer nano-composites containing MWCNT nano-fillers have been reported, providing great healing proficiency and recovery of physical properties [32,33].

Within this paper, the authors have synthesised by means of coaxial electrospinning, a microvascular network (MVN) of PAN nano-fibres filled with DCPD healing agent, in order to evaluate the healing ability on an aerospace matrix and composite material, by means of flexural tests. In addition, multiwalled carbon nanotubes (MWCNTs) were added to increase the healing capacity of the MVN.

## 2. Materials and Methods

### 2.1. Materials

Materials used to produce the microvascular self-healing system are shown in Table 1. A 10% PAN solution and a 10% DCPD solution were prepared using dimethylformamide (DMF) as a solvent for both solutions. For the preparation of PAN/DMF solution, 2 g of PAN was dissolved in 18 mL of solvent under continuous stirring at 400 rot/min and a temperature of 80 °C for six hours. The DPCD/DMF solution has been prepared by dissolving 1 g of DCPD in 9 mL solvent, using the same agitation rate and time, but at room temperature. MWCNTs have a purity greater than 95%, carboxyl-functionalized with a 10–30 nm in diameter and 30–50 nm in length. Materials presented in Table 1 were procured from Sigma-Aldrich (Sigma-Aldrich Chemie Gmbh, Munich, Germany).

### 2.2. Electrospinning Process

For the synthesis of self-healing microvascular network, a co-axial electrospinning equipment with a controlled temperature and humidity environment was used (IME Technologies, Waalre, The Netherlands).

**Figure 1:**
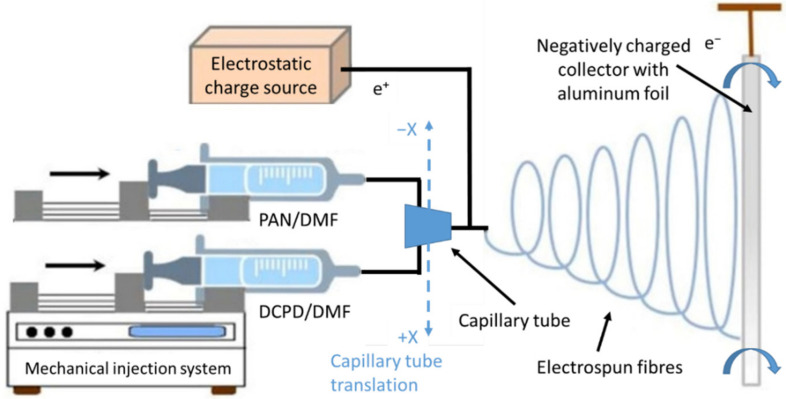
Schematic representation of the electrospinning process used to synthesise the self-healing microvascular network (adapted from [34]).

**Figure 2:**
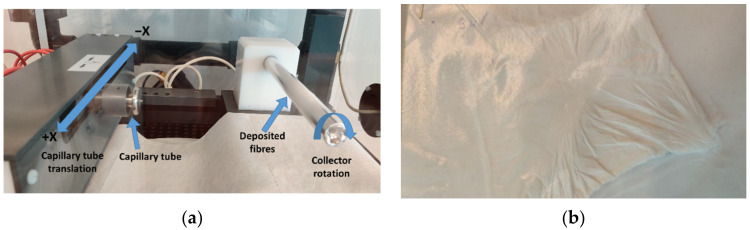
Images (**a**) during electrospinning process and of (**b**) synthesised microvascular network mesh.

The working and synthesis principle for the electrospinning process is presented in Figure 1. The two prepared solutions are mechanically injected using a controlled flow into a capillary tube. As the droplet is pushed away from the capillary tube, it is exposed to a high-voltage electrostatic field. A rotating cylindrical collector on the opposite side of the chamber is loaded with negative electrostatic charges that attract the polymer’s droplets. When these droplets are moving towards the collector, the solvent evaporates and on the collector are deposited only the polymer fibres, in a randomized network. An aluminium foil was placed on the collector for ease of nano-fibres extraction. The fibres are deposited in such a way due to the translation of the capillary tube parallel to the collector and due to electrostatic discharge, which oscillates the fibres prior to deposition on the collector. Synthesis parameters are described in Table 2 and in Figure 2 are presented images from the electrospinning process and with the electrospun fibres.

### 2.3. Chemical and Thermodynamic Analyses

The synthesised fibres were investigated by Fourier transform infrared spectroscopy (FT-IR), using a Nicolet i550FT-IR (Nicolet, Waltham, MA, USA) spectrometer. The apparatus is equipped with a high sensitivity DTGS detector, capable of measuring at a 4 cm^−1^ resolution within the 400 to 4000 cm^−1^ domain, creating a spectrum by co-adding 32 scans.

Two simultaneously thermal analyses were performed, including a thermogravimetric analysis (TGA) and differential thermal analysis (DTA), using a Shimadzu DTG-TA-50H equipment (Shimadzu Corporation, Kyoto, Japan) following a 10 °C/min heat ramp until 500 °C.

X-ray diffraction (XRD) has been conducted on the sample containing epoxy, nanofibers and carbon nanotubes, to evaluate the crystallinity of the samples. The analysis was performed on a Shimadzu XRD 6000 diffractometer (Shimadzu Corporation, Kyoto, Japan), with Ni filtered CuK α radiation (α = 1.5406 Å), 2 theta in the range 20–70°, with a scan step of 0.02° and a counting time of 0.6 s/step.

### 2.4. Microstructural Analysis

Scanning electron microscopy (SEM) analysis was performed to identify the network structure of the synthesized self-healing nano-fibres. The analysis was conducted using an Inspect F50 scanning electron microscope (FEI company, Brno, Czech Republic), at different magnifications.

### 2.5. Mechanical Tests

Flexural tests were performed to evaluate the self-healing capability of the synthesised microvascular network by means of three-point bending specimens. For the manufacturing of testing specimens, an epoxydic resin system was used at first (Resoltech 1050 resin and Hardener 1055S, Resoltech SAS, Rousset, France) to observe the behaviour of the healing system. Secondly, a pre-impregnated (prepreg) CFRP composite material (Hexcel Corporation, Stamford, CT, USA), was used for the manufacturing of composite specimens in order to evaluate if the microvascular network maintains its properties during manufacturing conditions (pressure, high temperature, vacuum) and to assess the self-healing ability after matrix cracking during constant loads. Multi-walled carbon nanotubes (MWCNT) were added due to their stronger reactions with the epoxydic matrix and faster vitrification mechanism of the whole system.

Specimens were fabricated in compliance to ISO 14125:2003 standard (Class IV specimens) [35]. The flexural tests were performed with a speed of 2 mm/min using Instron 3360 Series Universal Testing instrument (Instron, Norwood, MA, USA), with a force cell of 50 kN.

## 3. Results

### 3.1. Microstructural Analysis

**Figure 3:**
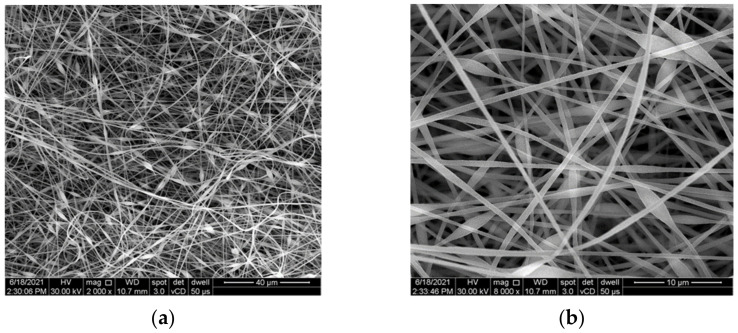

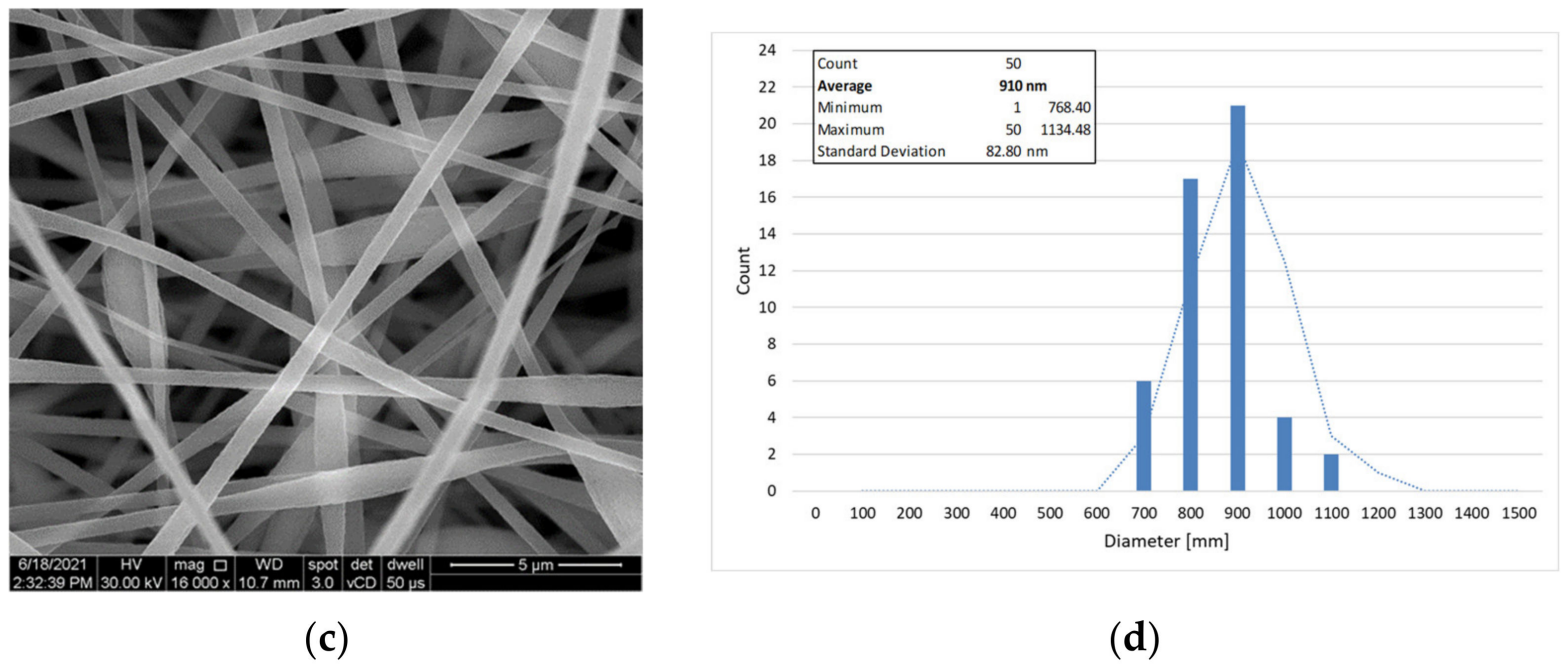
SEM images of initial microvascular network formed by electrospinning at different magnifications (**a**) 2000×, (**b**) 8000×, (**c**) 16,000×, and (**d**) the nano-fibres mean diameter.

**Figure 4:**
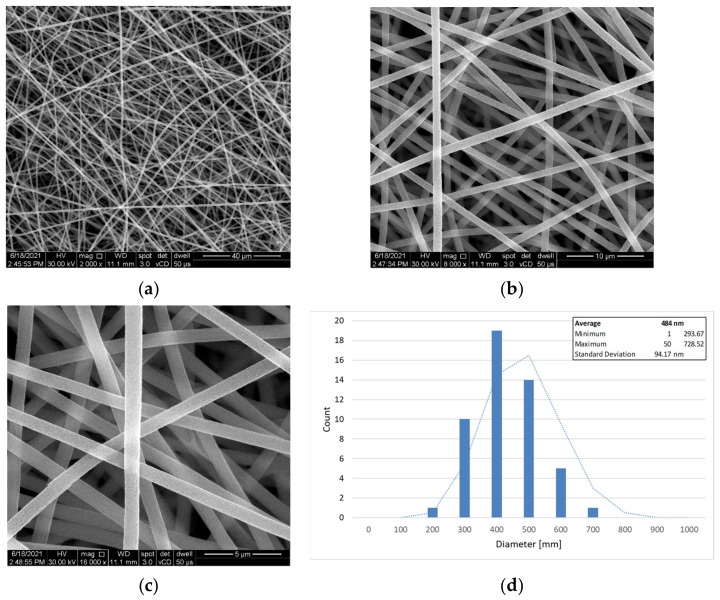
SEM images microvascular network formed by electrospinning after process improvement, at different magnifications (**a**) 2000×, (**b**) 8000×, (**c**) 16,000×, and (**d**) the nano-fibres mean diameter.

It can be seen from the SEM analyses in Figure 3 that the nano-fibre microvascular network was correctly synthesised, in a random arrangement due to the displacement of the capillary tube and the electrostatic discharge in the chamber. However, the appearance of beads is not ideal in forming of nano-fibres. Due to the electrostatic discharge and the high surface tensions caused by the solvent evaporation during electrospinning, the droplet is elongated and it modifies its geometry in a beads-on-string morphology. Increasing the electrostatic discharge from 13 kV to 14 kV and reducing the PAN/DMF:DCPD/DMF flow to 7:5 µL/min has proven to be successful in developing smooth, continuous and bead-free microvascular nano-fibres, as seen in Figure 4. Nano-fibre dimensions were between 700 nm and 1100 nm for the first electrospinning process. After process parameters correction, nanofibers with the mean diameter of 484 nm were obtained.

### 3.2. Chemical and Thermodynamic Analyses

**Figure 5:**
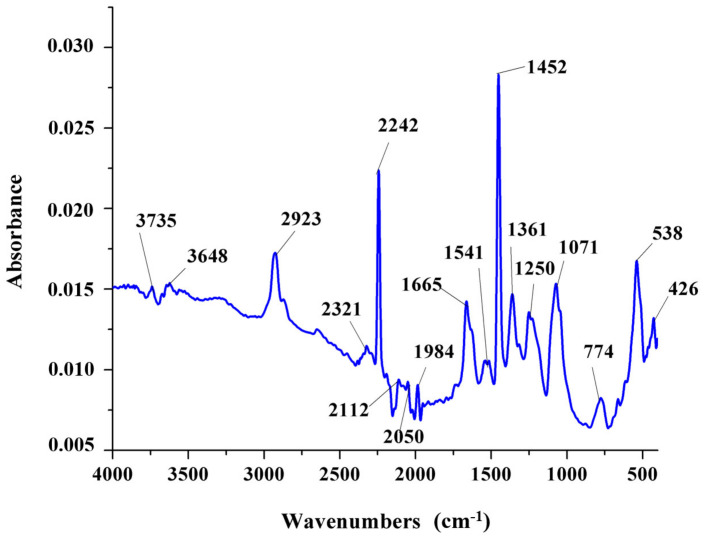
FT-IR analysis of PAN/DCPD electrospun microvascular network.

**Figure 6:**
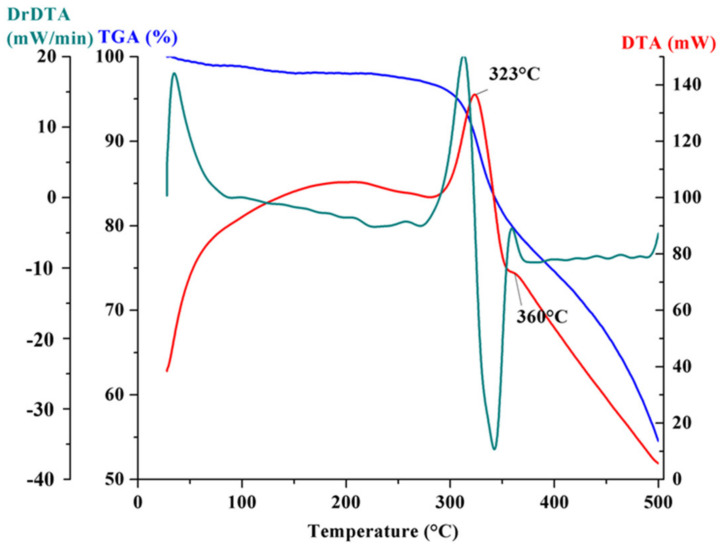
TGA/DTA analysis for the PAN/DCPD microvascular network.

The FT-IR spectrum recorded for the microvascular network is presented in Figure 5 and confirms the presence of both DCPD and PAN in the composition of the nanofibres. PAN show the characteristic absorption peaks of nitrile group at around 2240 cm^−1^ and the methylene groups in the range of 2930 cm^−1^, 1450 cm^−1^, and 1360 cm^−1^, while DCPD is characterized by the peak at 774 cm^−1^ attributed to its polymerization. Figure 6 shows the thermal analysis recorded on the PAN/DCPD microvascular network samples. The differential thermal analysis curve records the presence of two exothermic effects. The first effect with a maximum temperature of 323 °C is associated with the cyclizing effect of the nitrile group which causes a massive heat release and degradation of polymer chains, and the second exothermic effect (360 °C) can be associated to further dehydrogenation and crosslinking reactions.

**Figure 7:**
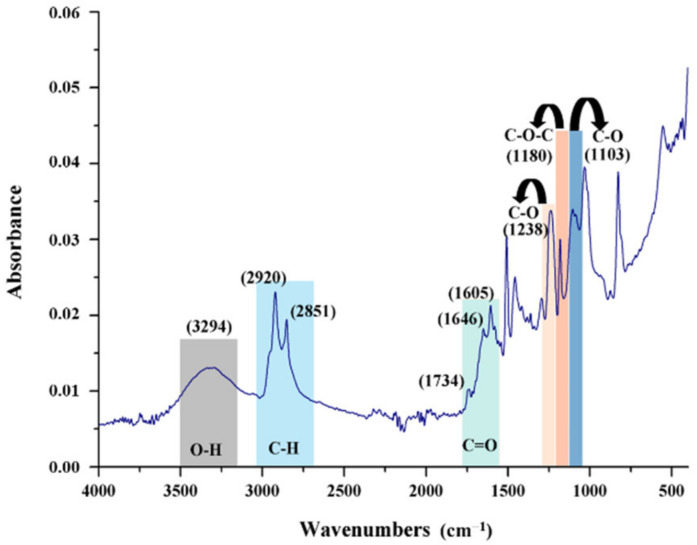
FT-IR characteristic peaks for MWCNT.

The peaks at 1600 cm^−1^ (Figure 7) shows the hexagonal structure of MWCNT, which can be attributed to the C=O bond stretching vibration mode. The 1300–1000 cm^−1^ region my be attributed to C–O vibration mode. The peaks found in the range of 3200–2800 cm^−1^ region can be attributed to asymmetric and symmetric stretching vibration of H–H bonds from a long alkyl chain, as the MWCNT are carboxyl functionalized. The appearance of these bands in the FTIR spectrum reveals the stability of MWCNTs.

The thermogravimetric curves of PAN/DCPD microvascular network showed a total of 45.50% mass loss of the nanofibers. In the first stage (27–300 °C) no large weight loss is noticed (4.31%), while the rate of weight loss in the second stage (300–350 °C) is much faster, being about 14.13% that is associated with the cyclization reaction observed on the DTA curve and in the third stage (350–500 °C), the mass loss is about 27.07% can be attributed to the oligomerization of nitrile groups in PAN. The rapid mass loss between 300–350 °C was observed (characteristic peak at 323 °C) and is correlated with the melting point of PAN shell and a slower one between 350–500 °C which is associated with the melting of DCPD core, as also seen in a previous work [11].

**Figure 8:**
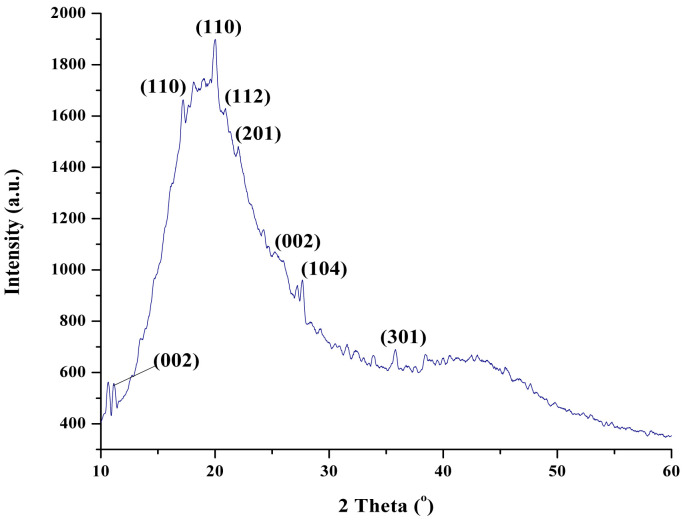
XRD pattern of sample containing epoxy/MVN/MWCNT.

According to the literature [36,37,38], adding CNT in the polymer matrix promotes the presence of a greater number of interfaces, favouring the interfacial polarization presence. Due to the non-crystalline structure of the epoxydic resin (polymeric material), it was difficult to observe carbon characteristic peaks. However, the XRD pattern presented in Figure 8 show the (002) index was observed close to 2θ = 10° and 2θ = 20°.

### 3.3. Mechanical Tests

#### 3.3.1. Epoxy System Specimens

**Figure 9:**
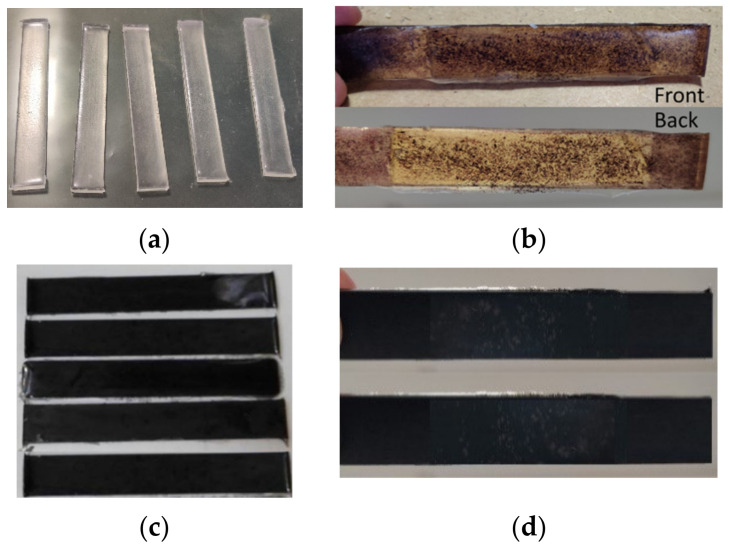
Images of (**a**) reference, (**b**) epoxy, microvascular network and Grubb’s catalyst, (**c**) epoxy and MWCNT and (**d**) epoxy, microvascular network, Grubb’s and MWCNT specimens.

Following the analyses and validation of the correct synthesis of the microvascular network, the evaluation of self-healing ability was conducted firstly by integration in an epoxydic matrix and secondly in fibre reinforced polymer (FRP) composite. Thus, six epoxy specimen sets (Table 3) were fabricated using a metallic mould and cured at 80 °C for 120 min. Each specimen set had a total of five tested specimens. Images of the cured specimens are presented in Figure 9. Following the curing process, all specimens were polished using a Metkon Forcipol 2V machine (Metkon Instruments Inc., Bursa, Turkey) and wet abrasive papers with 120 µm and 800 µm granulation.

Due to its thermoset nature, the matrix passes through a state of elastic deformation followed quickly by a plastic deformation that causes the material to break. Thus, it was decided that the reference specimens and specimens containing the MWCNTs be tested until rupture and the specimens containing the self-healing elements be tested until a 10% decrease in strength or until the material show a constant load (beginning of material degradation). This was considered in order to avoid the specimen rupture and thus the impossibility of evaluating the self-repair process, due to the nature of the matrix.

**Figure 10:**
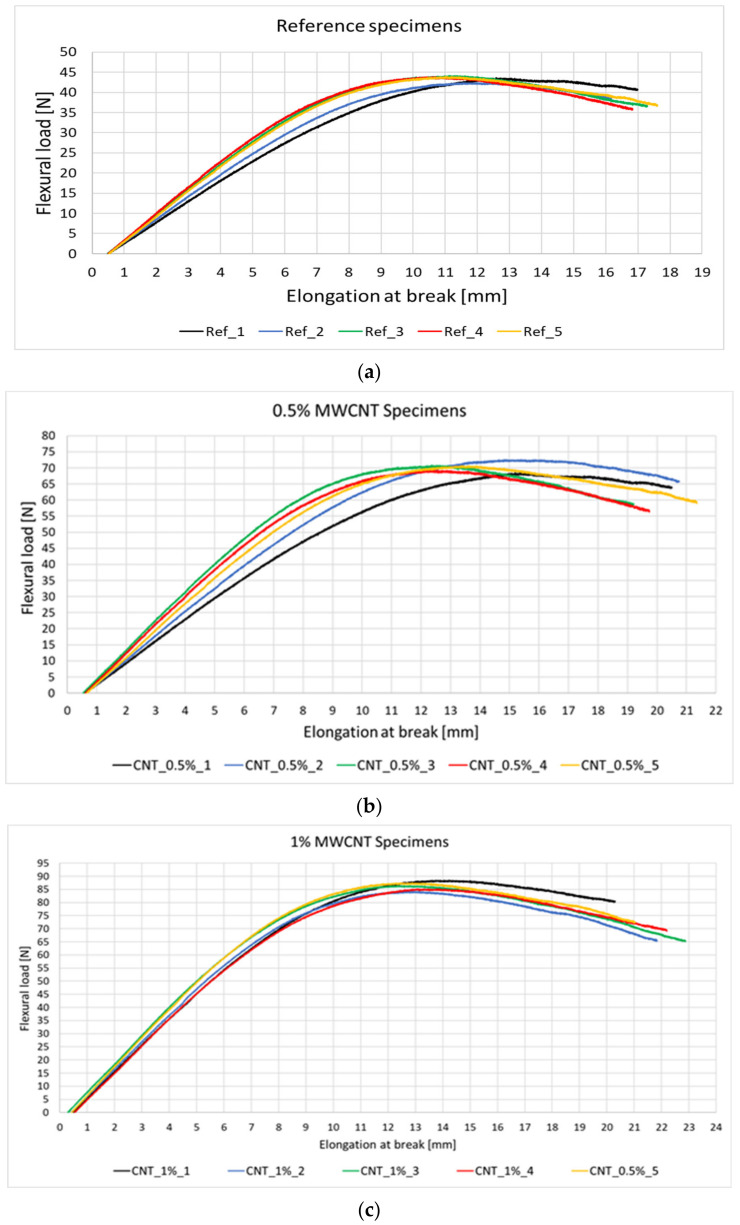
Flexural test results for the (**a**) reference, (**b**) 0.5%, and (**c**) 1% MWCNTs specimens.

The flexural tests for the reference and epoxy/MWCNTs specimens are presented qualitatively in Figure 10 and quantitatively in Table 4 as an average of the five specimens tested. It was observed that the addition of 0.5% MWCNTs resulted in an increase of 32% in flexural strength and 61% in flexural load when compared to the reference specimens. Further increasing the volume of MWCNT to 1% has doubled the flexural load and increased the flexural strength by 57% compared to the reference specimens. The elongation of specimens has also increased by 20% and 28%, respectively. Thus, the introduction of MWCNTs increases the flexural properties of the matrix and also its deformation capacity, representing a positive factor in the development of self-healing composites, as it delays the matrix failure, giving it more time for the healing agents to activate.

**Figure 11:**
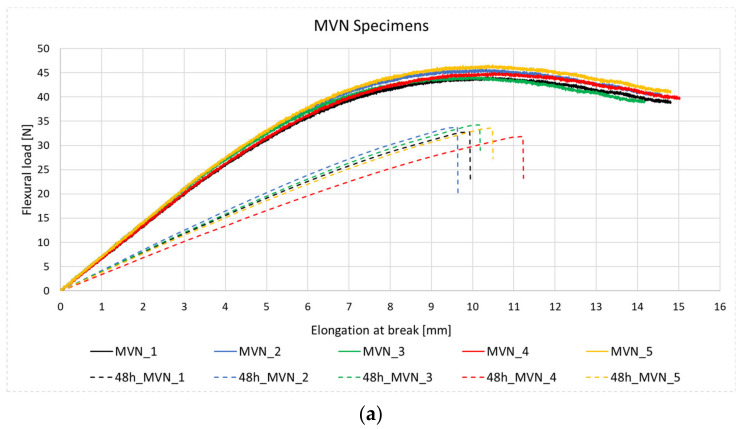

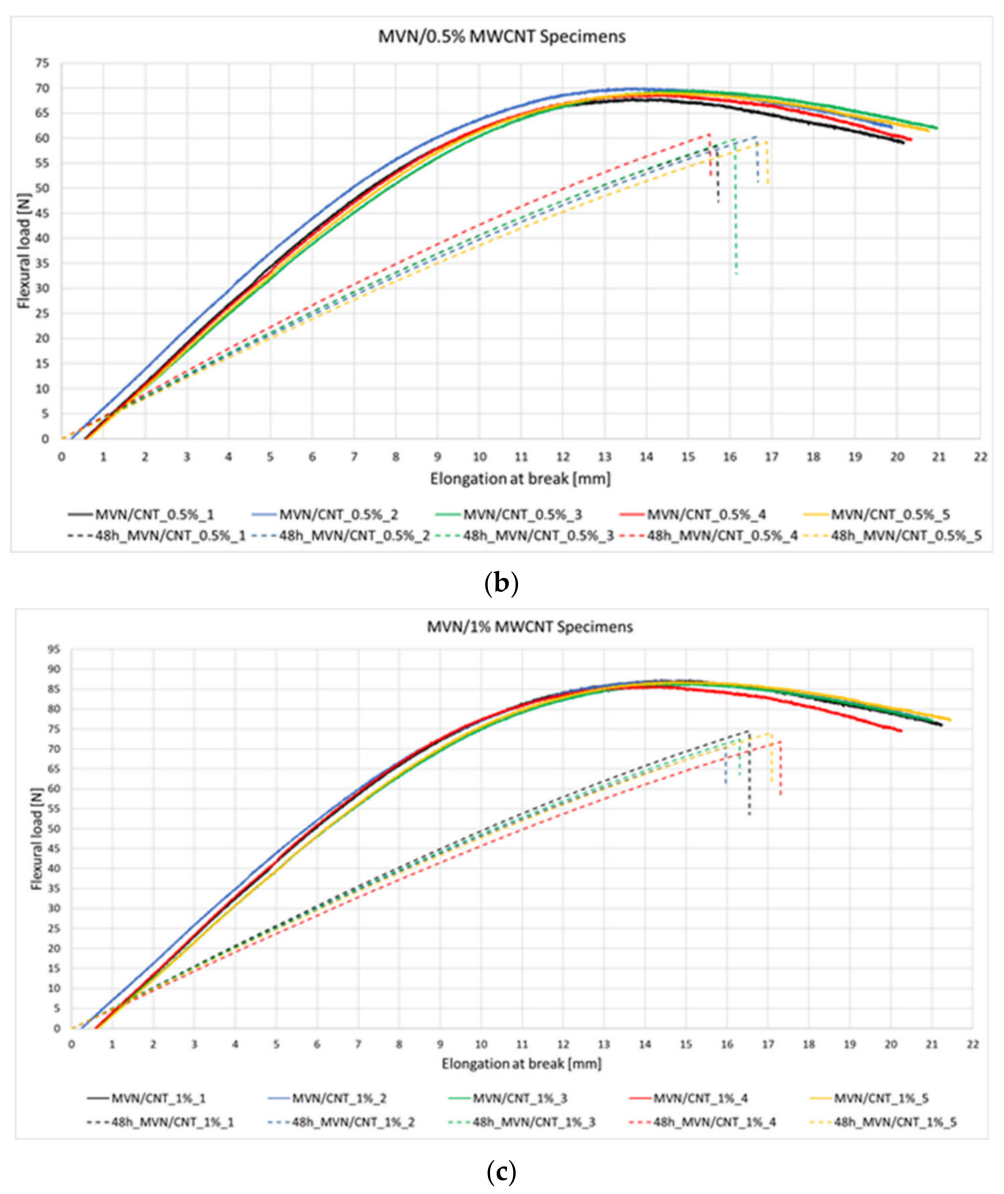
Flexural test results for the specimens containing the (**a**) self-healing system, (**b**) self-healing system and 0.5% MWCNT and (**c**) self-healing system and 1% MWCNT after first and second testing.

The addition of the MVN and Grubb’s catalyst did not influence the flexural properties of the matrix as compared to the reference specimens, and of the specimens containing epoxy/MWCNTs, as seen in Table 4. Following the first set of mechanical tests, the specimens containing the self-healing system were kept at 40 ± 1 °C for 48 h to release and activate the healing agents, prior to the second testing. Following the second testing of the specimens containing the self-healing system (Figure 11a), a 74% recovery in flexural load was observed compared to the initial test and 77% compared to the reference specimens. The maximum load peaks after the second test were found almost at the same deformation as in the initial test, suggesting the repair of the specimen.

For the specimens containing MVN/MWCNTs, the addition of 0.5% MWCNT lead to an 88% recovery of flexural strength and 87% recovery in flexural load (Figure 11b). Adding 1% MWCNT to the MVN system leads to 83% recovery in flexural strength and 84% in flexural load (Figure 11c). The failure points of retested specimens with MWCNTs are close to the stress values of the first test, indicating the repair of the matrix.

Considering the obtained results, it was concluded that although the 1% MWCNT addition enhances the mechanical performances of the matrix the most, it does not have the same characteristic of the healing efficiency. This can be explained by the fact that a bigger MWCNT volume is attached to the nano-fibres, making the rupture of the nano-fibres more difficult. Due to this aspect, it was considered to proceed further with only 0.5% MWCNT addition to the composite specimens.

**Figure 12:**
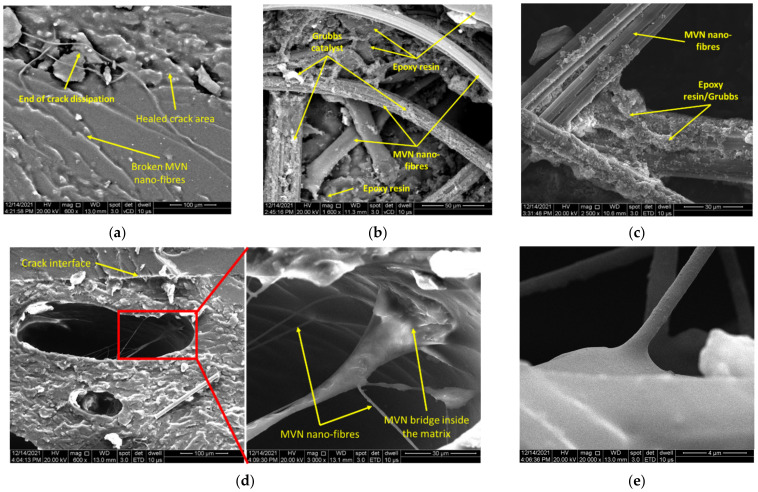
SEM analysis of the tested epoxy/MVN specimens, illustrating (**a**) crack bridging, (**b**,**c**) MVN nano-fibres bundles, (**d**) crack interface and nano-fibres bridge formation in the matrix, and (**e**) detailed view of a nano-fibre.

To confirm the healing process, samples were cut from the flexural area for SEM analyses. It was thus observed that the MVN behaves as a microvascular network, as illustrated in Figure 12. The crack propagation was also analysed. The healing morphology of the crack seems to modify near the crack end, by comparing Figure 12a to Figure 12d, as the MVN network is less dense near the end of the sample. Also, the nano-fibre network forms a bridging that aids the healing of the crack in its depth (Figure 12d).

**Figure 13:**
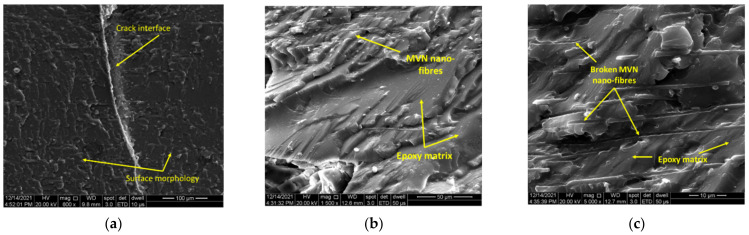

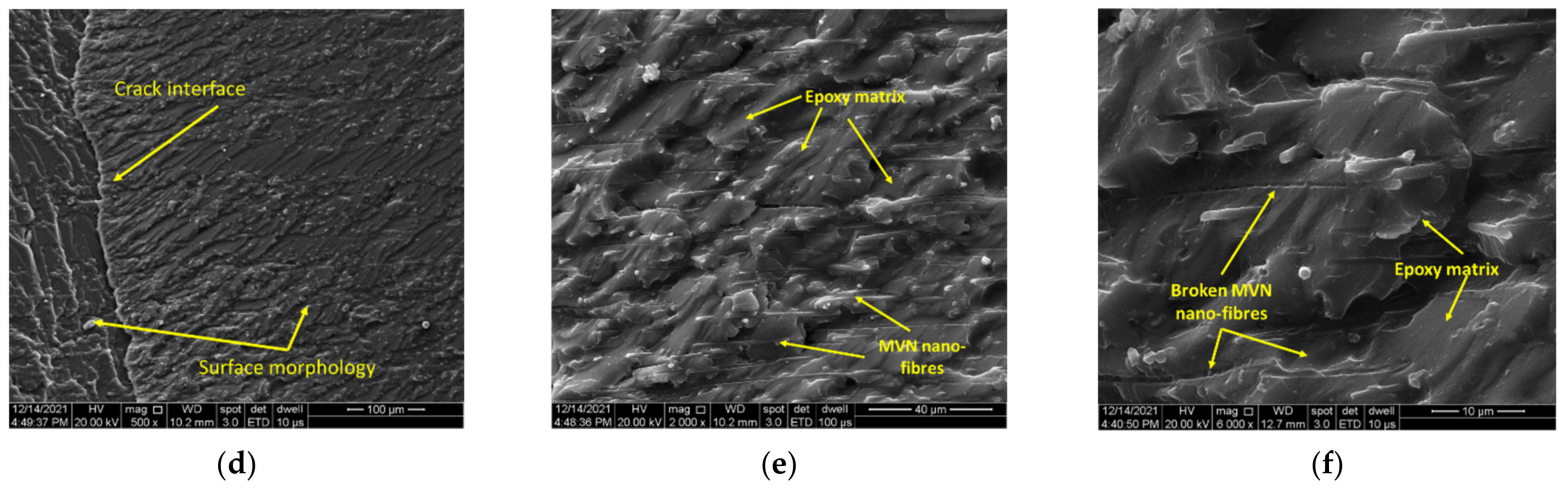
SEM analysis of the tested epoxy/MVN/MWCNT specimens, showing (**a**,**d**) crack interface, (**b**,**e**) an area from the crack plane and (**c**,**f**) detailed view of a crack plane for 0.5% and 1% MWCNT, respectively.

Samples were cut also from the MVN/MWCNTs specimens, subjected to SEM analysis and presented in Figure 13. Compared to the images shown in Figure 12, the crack healing is more pronounced for both 0.5% MWCNT (Figure 13a) and 1% MWCNT (Figure 13d). However, due to the high carbon content of the sample only the morphology of the crack plane and MVN nano-fibres could be observed. However, the presence of MWCNTs is validated through the obtained mechanical test results.

#### 3.3.2. FRP Composite Specimens

The self-healing system was embedded in CFRP composites to verify if it retains its healing properties after being subjected to elevated curing temperature and pressure (due to vacuum bag). Thus, four CFRP composite laminates were manufactured, as presented in Table 5. A total of 10 layers of prepreg was used for each composite lamina having the following curing process, 140 °C for 90 min, with a 3 °C/min. heating rate and 4 °C/min. cooling rate. Three layers of the MVN nano-fibres were applied between the middle plies of the lamina and coated with a thin layer of epoxydic matrix containing the Grubbs catalyst. For the MWCNT laminate, the nanotubes were applied between the same layers as for the MVN laminate. Same conditions applied for the MVN/MWCNT laminate.

**Figure 14:**
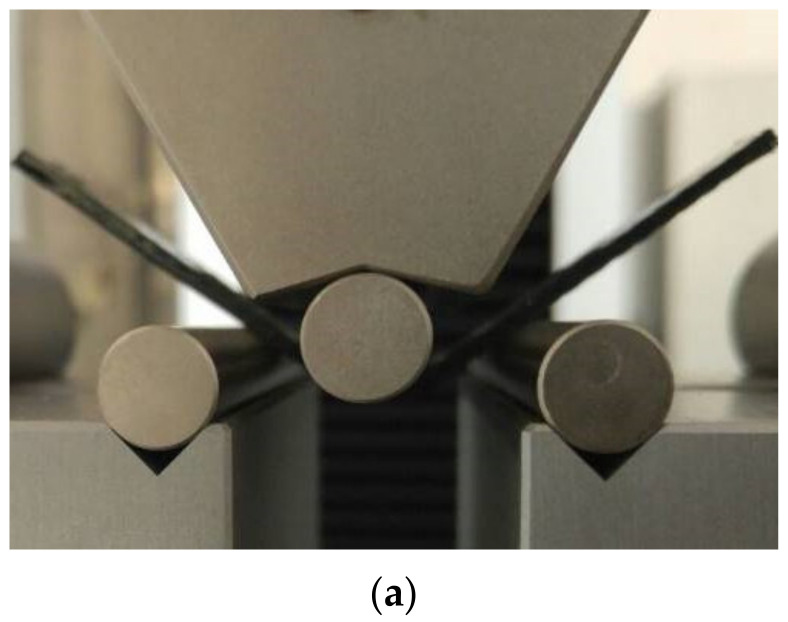

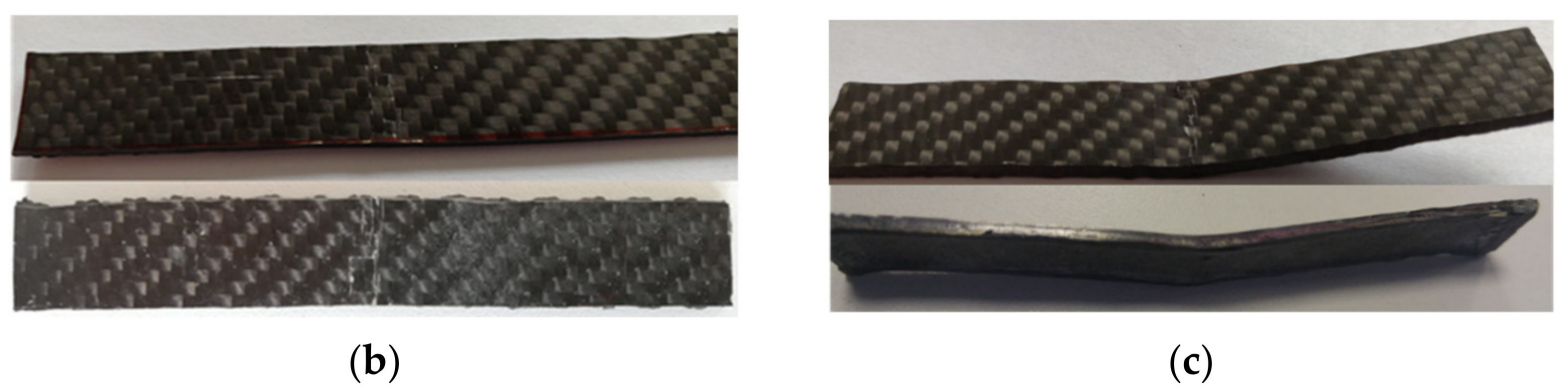
Images (**a**) during the testing campaign and of the tested specimens after (**b**) the first test and (**c**) the second test.

A total of 5 specimens were machined from each lamina and subjected to flexural tests, as depicted in Figure 14. Same testing conditions were applied as in the case of epoxy specimens. After the first test, all specimens were maintained for 48 h at a 40 ± 1 °C and retested.

**Figure 15:**
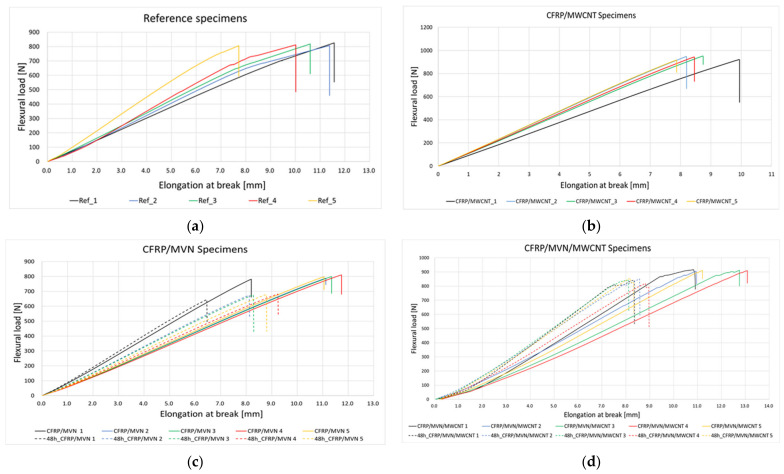
Flexural test results for the (**a**) CFRP reference specimens and (**b**) MWCNT specimens, (**c**) MVN specimens, and (**d**) MVN/MWCNT specimens after first and second testing.

Testing of the reference CFRP specimens was performed until rupture and showed a natural flexural behaviour, reaching a maximum of 814 N in flexural load and 832 MPa in flexural strength. As expected, the MWCNT enhances the specimens’ flexural properties, by 15%. The addition of MVN nano-fibres to the CFRP specimens has reduced the flexural load only by 2%. However, an increase of 12% was identified for the flexural strength. Thus, the self-healing MVN nano-fibres can act also as a reinforcement, as also mentioned in [16]. Adding both the nanofillers and the MVN nano-fibres to the CFRP specimen have demonstrated to increase both the flexural load and strength of the material, by 12%. After the retesting, the specimens containing MVN showed an 82% recovery in flexural load and an 88% recovery for flexural strength. The use of MWCNT nanofillers on the MVN specimen presented a 92.5% recovery in flexural load and a 93% in flexural strength. It can be said that by adding carbon nanotubes to the self-healing system has drastically increased the healing ability of the material. The results are presented qualitatively in Figure 15 and quantitatively in Table 6.

**Figure 16:**
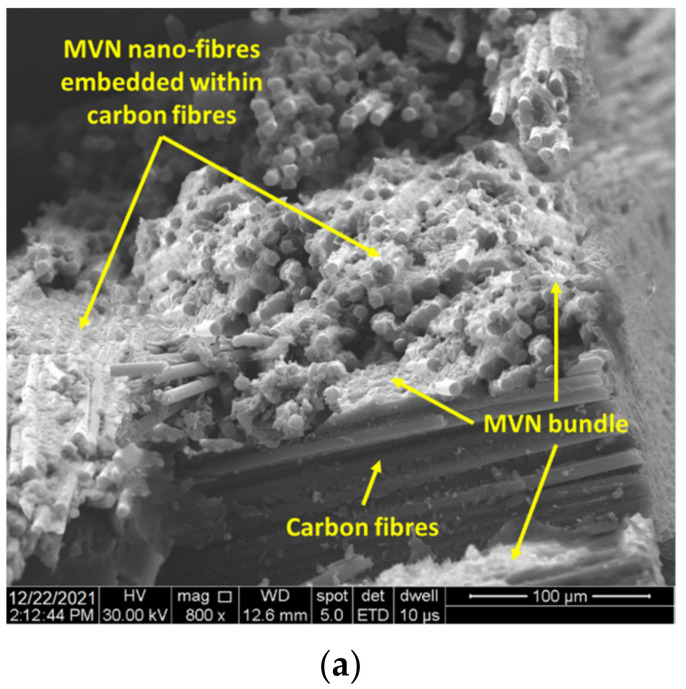

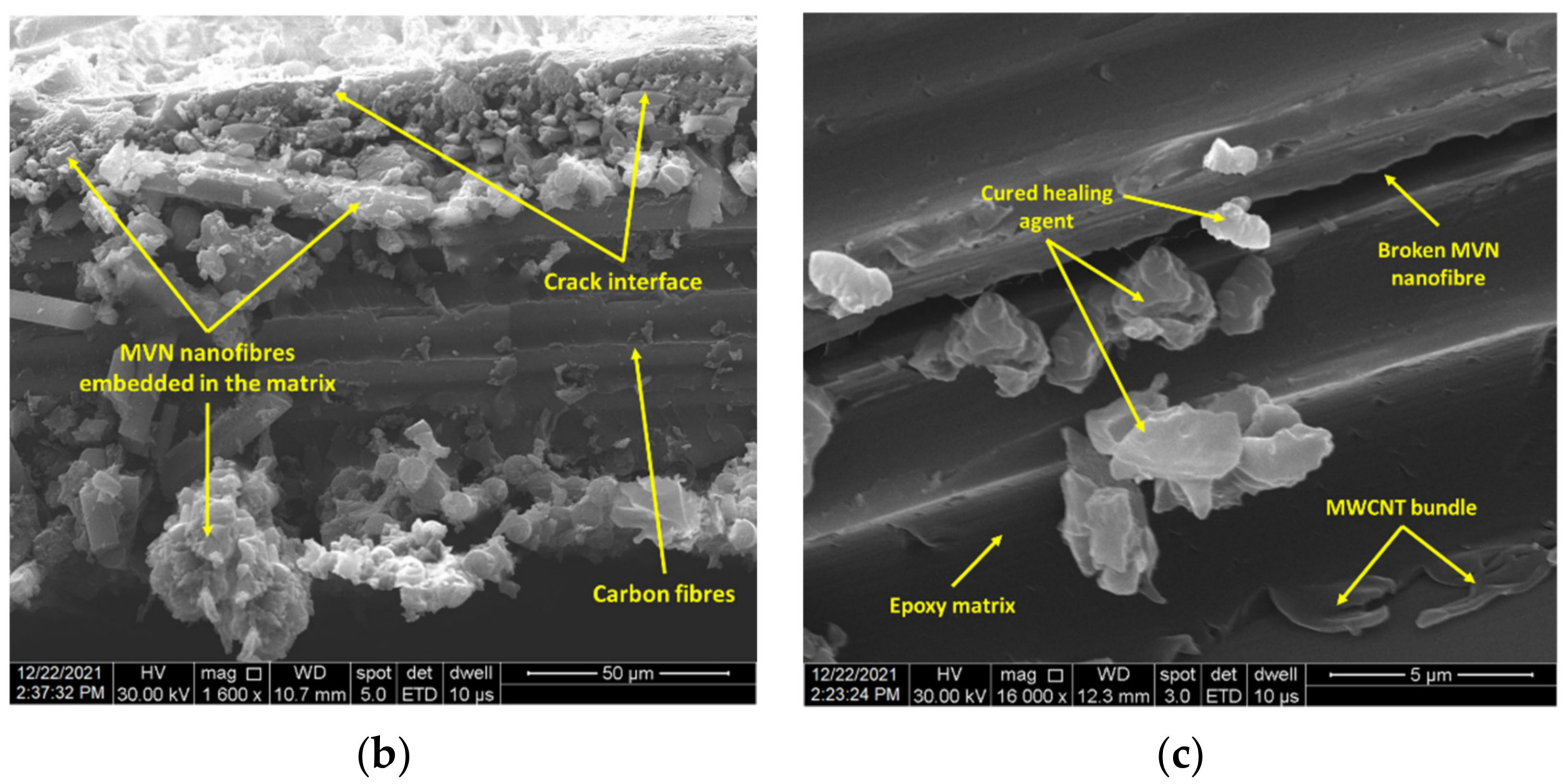
SEM analysis of CFRP specimens containing MVN and MVN/MWCNT at different magnification levels, at (**a**) 800×, (**b**) 1600×, (**c**) 16,000×.

To further confirm the CFRP matrix healing, samples were cut from the flexural area and subjected to SEM analysis, presented in Figure 16. Unlike the resin specimens, the crack interface was more difficult to be observed due to the high carbon content and contrast. Figure 16 presents the morphology of the sample, showing the MVN nano-fibres embedded in the epoxydic matrix and placed between the composite layers. Also, after a section cut through the flexural area and a higher magnification level, Figure 16b illustrates the crack interface. A brighter contrast at the interface suggests the presence of the hardened healing agent. At higher magnifications, both the catalyst and MWCNT could be observed attached to the epoxydic matrix and near broken MVN nano-fibre (Figure 16c). Future investigations envisage the use of GFRP to ease the observation of the crack healing, nanofillers and microvascular network, due to the low carbon content which affects the image processing.

## 4. Discussion

Given that most studies aim to investigate the use of microcapsules and hollow tubes as encapsulation methods for healing agents in polymer composites, this paper addresses the development and evaluation of a microvascular nano-fibre network, fabricated by coaxial electrospinning. Thus, a PAN/DCPD nano-fibre network has been obtained and used for the evaluation of its healing ability for aerospace graded materials, an epoxydic matrix and a pre-impregnated carbon fibre, respectively. The morphological studies by SEM revealed that the obtained nano-fibre network present a high density of beads on fibres. As this is not ideal for the electrospinning processing, the fabrication parameters were modified in accordance with suggestions provided in [17], which led to a smooth, uniform and bead-free nano-fibres, which are comparable to the ones described in other studies [1,10,16,21,39,40,41]. FT-IR analysis confirm the presence of PAN nano-fibres specific peaks and the presence of DCPD healing agent, as also identified in [42] and [11], respectively.

The healing ability was appraised by means of three-point bending tests for specimens containing the MVN nano-fibres and MVN nano-fibres with MWCNTs nanofillers to improve the healing mechanism. It was observed that the addition of 0.5% MWCNT nanofillers has improved the healing ability of the epoxydic matrix, as also validated by SEM, illustrating the healing crack interface and bridging of the MVN nano-fibres. CFRP composites specimens were also investigated using the same nano-fibres and nanofillers, concluding the same healing process ability. Also, the addition of the MVN nano-fibres increased the flexural strength of the composite specimen, concluding that besides the healing ability, the nano-fibres network acts also as a reinforcement.

Future work envisages the use of GFPR composite to have a better overview of the crack area and healing process, due to the less carbon content. Moreover, the analysis of an electric impulse trigger to initiate the healing process is also foreseen, due to the electro-mechanical properties of the MWCNT nanofillers.

## 5. Conclusions

To pursue the self-healing properties of microvascular systems incorporated in polymeric composite materials, specific specimens were fabricated and subjected to three-point mechanical bending tests. Also, nanofiller elements (MWCNTs) were used to increase the molecular reactions between the free radicals of the repair agent (DCPD) and the epoxydic matrix. The use of carbon nanotubes also improves the electrical conductivity of the material, which once transformed into thermal conductivity, speeds up the repair process. This could represent a major benefit for aerospace structures, especially at aircraft control surfaces (slats, spoilers, aileron, flaps) and fuselages. As most of these structures are electronically manoeuvred, electric impulses can trigger the healing mechanism within the composite.

The use of a microvascular self-healing system can be extremely beneficial in manufacturing thermosetting polymeric composite materials, as it has no negative effect on the nominal properties of the epoxydic matrix material and can be applied between each layer of the laminate, without causing delamination. This is due to the fact that the matrix encloses the microvascular system during the curing process, and it can also attach to the reinforcing fibres, as seen in the present paper.

## Figures and Tables

**Table 1:** Materials used for the microvascular network synthesis process.

Material	Molecular Formula	Physical Properties	Role in the Synthesis
PAN	(C_3_H_3_N)_n_	White crystalline powder. Melting point at 300 °C.	Formation of nano-fibres shell.
DCPD	C_10_H1_2_	Gel-like (room temperature). Melting point at 32.5 °C.	Nano-fibres core, microvascular self-healing agent.
DMF	C_3_H_7_NO	Colourless liquid solution, strong ammonia odour. Melting point at −61 °C.	Used as solvent for PAN and DCPD.
Grubb’s catalyst	C_43_H_72_C_l2_P_2_Ru	Purple crystalline powder. Melting point at 153 °C.	Used for activation of self-healing agent.

**Table 2:** Microvascular network synthesis parameters.

Parameter	Value
Chamber humidity	35%
Chamber temperature	25 °C
Distance between capillary tube and collector	160 mm
Capillary tube translation	140 mm (−70 mm/+70 mm)
Capillary tube translation speed	3 mm/s
PAN/DMF: DCPD/DMF flow	9:4.5 µL/min
Collector rotation speed	150 rot/min
Applied voltage	17 kV

**Table 3:** Epoxy resin specimen to be tested.

Specimen Sets	Specimen Type	Relevance of Test
1	Neat epoxy	Reference specimens
2	Epoxy system with 0.5% MWCNT	Evaluate the MWCNT influence over the mechanical properties of epoxydic matrix
3	Epoxy system with 1% MWCNT	
4	Epoxy system with MVN and Grubb’s catalyst	Evaluating the mechanical properties and self-healing ability of matrix with the introduction of new elements
5	Epoxy system with MVN, Grubb’s catalyst and 0.5% MWCNT	
6	Epoxy system with MVN, Grubb’s catalyst and 1% MWCNT	

**Table 4:** Average results for the three-point bending tested specimens.

Specimen Type	Maximum Flexural Strength, σ_m_ (MPa)	Flexural Load at Break, F (N)	Elongation at Break, ε (mm)	Flexural Modulus, E [MPa]	Absorbed Energy, AE [J]
Epoxy Reference	65.17	43.59	16.98	3812.35	4671.30
Epoxy/0.5% MWCNT	85.78	70.37	20.31	6333.83	7056.04
Epoxy/1% MWCNT	102.12	86.43	21.65	7717.50	7118.11
Epoxy/MVN/Grubbs	63.26	45.19	14.68	5476.00	3294.30
Epoxy/MVN/Grubbs (48 h)	41.76	33.28	10.29	3231.54	3263.02
Epoxy/MVN/Grubbs/0.5% MWCNT	89.12	69.15	20.43	6151.89	6502.78
Epoxy/MVN/Grubbs/0.5% MWCNT (48 h)	78.02	60.15	16.17	4456.31	4246.08
Epoxy/MVN/Grubbs/1% MWCNT	99.39	86.77	20.99	7714.41	8143.10
Epoxy/MVN/Grubbs/1% MWCNT (48 h)	82.70	72.66	16.65	4939.92	5129.15

**Table 5:** Composite specimens to be tested.

Laminate No.	No. of Specimens	Specimen Type
1	5	CFRP Reference
2	5	CFRP/0.5% MWCNT
3	5	CFRP/MVN
4	5	CFRP/MVN/0.5% MWCNT

**Table 6:** Average results for all three-point bending CFRP composite specimens.

Specimen Type	Maximum Flexural Strength, σ_m_ (MPa)	Flexural Load at Break, F (N)	Flexural Strain, ε (mm)	Flexural Modulus, E [MPa]	Absorbed Energy, AE [J]
CFRP Reference	832.25	813.99	10.26	45,276.86	95,181.85
CFRP/MWCNT	947.18	936.56	8.64	48,238.86	101,408.67
CFRP/MVN	934.45	795.18	10.69	44,515.16	94,025.40
CFRP/MVN/MWCNT	942.96	910.48	11.78	47,392.57	99,629.57
CFRP/MVN (48 h)	730.24	671.01	9.02	49,635.25	99,540.61
CFRP/MVN/MWCNT (48 h)	878.86	843.09	8.48	51,073.96	107,368.65

## Data Availability

The data presented in this study are available on request from the corresponding author. Due to the contract agreement between COMOTI and the funding agency, the research activities and data presented in this paper will be presented in an Executive Summary Report and will be available for public use, after project closure.

## Abbreviations

CFRP	Carbon Fibre Reinforced Polymers
CNT	Carbon Nanotubes
DCPD	Dicyclopentadiene
DMF	Dimethylformamide
DTA	Differential Thermal Analysis
FRP	Fibre Reinforced Polymer
FTIR	Fourier Transform Infrared
GFRP	Glass Fibre Reinforced Composite
MVN	Microvascular Network
MWCNT	Multi Walled Carbon Nanotubes
PAN	Polyacrylonitrile
PCM	Polymeric Composite Materials
PMMA	Poly(methyl Methacrylate
SAN	Styrene Acrylonitrile
SEM	Scanning Electron Microscopy
TGA	Thermogravimetric Analysis
